# DNA Damage in Moderate and Severe COVID-19 Cases: Relation to Demographic, Clinical, and Laboratory Parameters

**DOI:** 10.3390/ijms251910293

**Published:** 2024-09-24

**Authors:** Tigran Harutyunyan, Anzhela Sargsyan, Lily Kalashyan, Naira Stepanyan, Rouben Aroutiounian, Thomas Liehr, Galina Hovhannisyan

**Affiliations:** 1 Laboratory of General and Molecular Genetics, Research Institute of Biology, Yerevan State University, Alex Manoogian 1, Yerevan 0025, Armenia; tigranharutyunyan@ysu.am (T.H.); angela.sargsyan@ysu.am (A.S.); lilikalashyan@ysu.am (L.K.); genetik@ysu.am (R.A.); galinahovhannisyan@ysu.am (G.H.); 2 Department of Genetics and Cytology, Yerevan State University, Alex Manoogian 1, Yerevan 0025, Armenia; 3 National Center for Infectious Diseases, Arno Babajanyan 21, Yerevan 0064, Armenia; nsstepanyang@gmail.com; 4 Jena University Hospital, Institute of Human Genetics, Friedrich Schiller University, Am Klinikum 1, D-07747 Jena, Germany

**Keywords:** COVID-19, comet assay, DNA damage, SARS-CoV-2 virus

## Abstract

The ability of the SARS-CoV-2 virus to cause DNA damage in infected humans requires its study as a potential indicator of COVID-19 progression. DNA damage was studied in leukocytes of 65 COVID-19 patients stratified by sex, age, and disease severity in relation to demographic, clinical, and laboratory parameters. In a combined group of COVID-19 patients, DNA damage was shown to be elevated compared to controls (12.44% vs. 5.09%, *p* < 0.05). Severe cases showed higher DNA damage than moderate cases (14.66% vs. 10.65%, *p* < 0.05), and males displayed more damage than females (13.45% vs. 8.15%, *p* < 0.05). DNA damage is also correlated with international normalized ratio (INR) (r = 0.471, *p* < 0.001) and creatinine (r = 0.326, *p* < 0.05). In addition to DNA damage, severe COVID-19 is associated with age, C-reactive protein (CRP), and creatinine. Receiver operating characteristic analysis identified age, INR, creatinine, DNA damage, and CRP as significant predictors of disease severity, with cut-off values of 72.50 years, 1.46 s, 78.0 µmol/L, 9.72%, and 50.0 mg/L, respectively. The results show that DNA damage correlates with commonly accepted COVID-19 risk factors. These findings underscore the potential of DNA damage as a biomarker for COVID-19 severity, suggesting its inclusion in prognostic assessments to facilitate early intervention and improve patient outcomes.

## 1. Introduction

The global Coronavirus Disease 2019 (COVID-19) pandemic, caused by the severe acute respiratory syndrome coronavirus 2 (SARS-CoV-2), has had a huge impact, with more than 775 million cases and over 7 million deaths reported worldwide as of 14 July 2024 [1]. The World Health Organization (WHO) declared an end to the COVID-19 global health emergency on 5 May 2023 [2,3,4]. However, SARS-CoV-2 still poses a global threat due to the development of new viral variants that might escape immunity [5]. According to the COVID-19 epidemiological update as of 17 June 2024, WHO is currently tracking several SARS-CoV-2 variants: three variants of interest: EG.5 (nickname Eris, descendant of Omicron), BA.2.86, and JN.1; and four variants under monitoring: JN.1.7, JN.1.18, KP.2, and KP.3 [6]. One of the currently circulating Omicron subvariants, EG.5, has exhibited an increased effective reproductive rate, prompting concerns about its contagiousness and immune evasion capabilities [7].

COVID-19 is characterized by an unpredictable and extremely variable disease course ranging from asymptomatic cases to severe illness. Until now, significant gaps remain in our knowledge about the causes of mild, moderate, or severe clinical courses, as well as the long-term consequences of this disease. The broad spectrum of clinical manifestations of COVID-19 requires the search for the identification of predictive molecular markers, which currently mainly focus on clinical and laboratory parameters.

According to Grand [8], until recently, the relationship between the virus and human genome stability, as well as the DNA damage response (DDR), were poorly studied. However, it is now evident that SARS-CoV-2 damages host cell DNA and intricately interacts with the cellular DDR [9]. COVID-19 was shown to be linked with aberrant activation of DDR [10,11,12].

DNA damage studies were performed in COVID-19 patients in different tissues like blood [13,14,15,16,17], lung [18] and cardiac [19] tissue samples (including such from fatal COVID-19 cases [18]), as well as lung of SARS-CoV-2-infected hACE2-mouse [12]. There are studies both with [14,17] and without [13,15,16] stratifying the studied patients according to the severity of COVID-19. SARS-CoV-2-induced DNA damage was also analyzed in infected cell lines [12,20,21].

The effects of SARS-CoV-2 on the host genome were analyzed with alkaline [12,13,14,15,17,22] or neutral [16] versions of the comet assay using Tail DNA (%) [13,14,15,17,22], Tail Moment, and Olive Tail Moment [12] as endpoints. DNA damage has also been studied using micronucleus tests [21,22], γH2AX (marker of double-strand break (DSB)) and pRPAS4/8 (marker of SSB) foci [12,18,19,20].

Despite ongoing research, there are still significant gaps in our understanding of the relationship between disease progression and genetic markers. One reason for this is the wide variation in the designs of the studies, which makes it difficult to interpret them together.

Summarizing the available publications on DNA damage in COVID-19 Grand [8] notes that “Because almost all the information discussed here has been published recently and may be preliminary in a few instances, in some cases there are inconsistencies and divergent views, which will need further evaluation for verification in the future; however, it is hoped that the case has been made that the DDR is an important target for SARS-CoV-2”.

It is known that DNA damage and impaired repair mechanisms foster genome instability and are involved in several chronic diseases. One possible consequence of DNA damage in COVID-19 patients may be cardiac problems [19]. In addition, SARS-CoV-2 proteins can elevate genomic instability and pose potential oncogenic activity via degradation of the co-suppressors retinoblastoma protein (pRB) and p53 [23].

The aim of this study was to evaluate DNA damage using the alkaline comet assay and assign the severity of DNA damage to disease severity in COVID-19 patients. In addition, possible correlations between DNA damage in blood and laboratory parameters in COVID-19 patients were evaluated.

## 2. Results

### 2.1. Demographic, Clinical, and Laboratory Parameters of COVID-19 Patients

Sixty-five hospitalized patients with COVID-19 confirmed by quantitative reverse transcription polymerase chain reaction (RT-qPCR) were divided into moderate (30 cases, 46.15%) and severe (35 cases, 53.85%) infection groups (Table 1).

In the moderate group, 52.8% were women and 47.2% were men. In the group of severely ill patients, 55.2% were women and 44.8% were men. Thus, the sexual composition of the two groups was not significantly different.

Statistically significant differences in demographic and clinical characteristics of male and female patients were not identified (*p* > 0.05); therefore, the data are presented in combined groups (Table 1).

The patients in the severe group were older (median age 72 years, with interquartile range = IQR of 63.0–70.0) than the patients in the moderate group (median age 62 years, IQR 44.5–69.5) (*p* < 0.05).

At least one comorbidity was recorded in 84.61% of the patients. The most common comorbidities, arterial hypertension, diabetes mellitus, and heart disease, are equally prevalent in moderate and severe groups of patients. Stroke, pneumocystis pneumonia, bronchial asthma, thyroid disease, chronic obstructive pulmonary disease, varicose veins of the lower extremities, epilepsy, and gastrointestinal bleeding accounted for less than 4% of the moderate and severe cases. There was no difference in the level of comorbidity between patients and controls.

The elevated body mass index (BMI) indicates obesity in both groups of patients according to the criteria for Asian and South Asian populations [24]. A significant difference between the BMI of the control group and COVID-19 patients was identified (*p* < 0.05). The BMI in the severe group is higher than in the moderate group, without a significant difference between them (29.1 (25.3–31.6) versus 26.1 (22.5–29.5)).

Differences in levels of smoking status and alcohol consumption between patients with moderate and severe infection, as well as between patients and the control group, were not found.

Included in this study were crucial biomarkers of COVID-19, including hematological parameters (white blood cells (WBC), neutrophils (NEU), lymphocytes (LYM), neutrophil to lymphocyte ratio (NLR), platelets (PLT)), inflammation parameters (C-reactive protein (CRP), and procalcitonin (PCT)), coagulation parameters (D-dimer, international normalized ratio (INR), activated partial thromboplastin time (APTT), and fibrinogen), and end-organ injury biomarkers, namely renal (creatinine) and hepatic (alanine transaminase (ALT), and aspartate transferase (AST)).

Laboratory findings of patients with moderate and severe COVID-19 are shown in Table 2. CRP, PCT, D-dimer, INR, and fibrinogen were higher than reference values in both moderate and severe patients. Lymphopenia was observed in the severe group. The median values of other laboratory parameters were within normal limits, with pathological deviations observed only in individual patients. Patients with severe COVID-19 had significantly higher CRP and creatinine and a longer duration of INR and hospital stay compared to patients in the moderate group (*p* < 0.05).

Laboratory parameters were analyzed in COVID-19 patients in the context of age- and sex-related changes (Table 2 and Table 3). Both men and women with COVID-19 were older in the severe than in the moderate group. Men with severe versus moderate illness had higher BMI and CRP. Women with severe versus moderate disease had higher INR levels and longer hospital stays. The only difference found between men and women with severe COVID-19 was that the procalcitonin (inflammation index) was higher in men than in women (Table 3).

We categorized patients into two age groups, namely adults (15 to 64 years) and elderly (65 years or older). In the adult age group, patients with severe forms of COVID-19 have a longer hospital stay compared with the moderate form. In the elderly age group, in addition to a longer length of hospital stay, CRP and INR were also higher in patients with severe disease compared with moderate disease. Finally, fibrinogen levels and length of hospital stay are higher in the elderly age group compared with adults (Table 4).

### 2.2. DNA Damage in COVID-19 Patients

**Figure 1:**
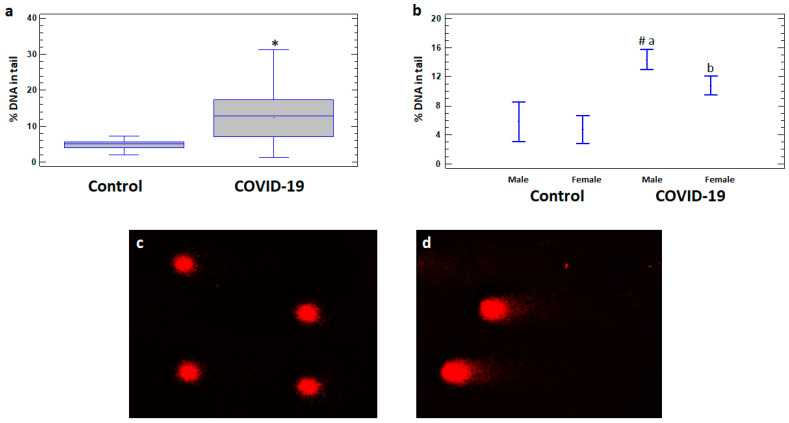
% DNA in tail in leukocytes of healthy controls and COVID-19 patients. (**a**) % DNA in tail in leukocytes of control and COVID-19 patient groups. (**b**) % DNA in tail in leukocytes of male and female subgroups of healthy control and COVID-19 patients. Examples of DNA comets obtained by comet assay in peripheral blood leukocytes of (**c**) a healthy control subject and (**d**) a COVID-19 patient. The values are given as means ± SE = standard error. * *p* < 0.05—significant difference compared to control; ^a^ *p* < 0.05—significant difference compared to control in males; ^b^ *p* < 0.05—significant difference compared to control in females; ^#^ *p* < 0.05—significant difference compared to female patients.

There are no significant differences in DNA damage levels between COVID-19 patients with and without comorbidities, so the data on these subgroups are analyzed together. The levels of DNA damage expressed as% DNA in tail of comets in leukocytes were significantly higher in the total group of COVID-19 patients (12.44 ± 0.79%) as well as in male (14.36 ± 0.82%) and female (10.79 ± 1.24%) patients compared to the corresponding controls (5.09 ± 0.43%, 5.80 ± 1.22%, and 4.79 ± 0.23%, respectively) (Figure 1a,b). Moreover, the levels of DNA damage were significantly higher in male patients compared to female patients (*p* < 0.05) (Figure 1b).

**Figure 2:**
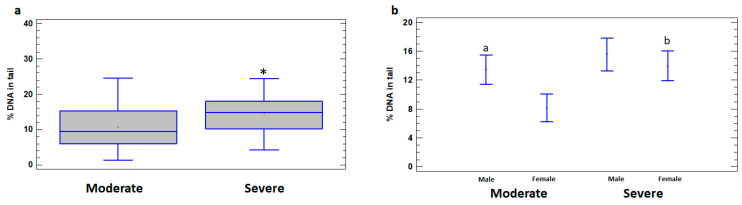
% DNA in tail in leukocytes of male and female patients with moderate and severe COVID-19. (**a**) % DNA in tail in leukocytes of moderate and severe COVID-19 patient groups. (**b**) % DNA in tail in leukocytes of male and female subgroups of patients with moderate and severe COVID-19. The values are given as means ± SE. * *p* < 0.05—significant difference compared to moderate patients; ^a^ *p* < 0.05—significant difference compared to moderate female patients; ^b^ *p* < 0.05—significant difference between severe and moderate female patients.

Higher levels of DNA damage in leukocytes of the total group of severely ill patients (14.66 ± 1.15%) compared to patients with moderate illness (10.65 ± 1.01%) were revealed (*p* < 0.05) (Figure 2a). In addition, the levels of DNA damage were significantly higher in leukocytes of severely ill female patients (13.94 ± 1.85%) compared to females with moderate illness (8.15 ± 1.44%) (*p* < 0.05). The differences between the severe and moderate cases in male patients do not reach statistical significance (*p* > 0.05) (Figure 2b). In the group of patients with moderate illness, % DNA in the tail was higher in leukocytes of males (13.45 ± 1.09%) compared to females (8.15 ± 1.44%) (*p* < 0.05); however, in the severe group, the difference between the sexes was not significant (*p* > 0.05) (Figure 2b).

**Figure 3:**
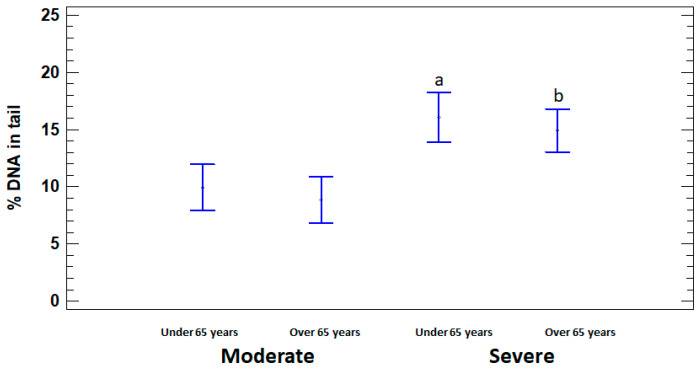
% DNA in tail in leukocytes of adults (under 65 years) and elderly (65 years or older) patients with moderate and severe COVID-19. The values are given as means ± SE. ^a^ *p* < 0.05—significant difference compared to moderate patients under 65 years; ^b^ *p* < 0.05—significant difference compared to moderate patients over 65 years.

In order to assess the dependence of DNA damage on age, the patients with moderate and severe illness were divided into two subgroups, namely adults (15 to 64 years) and elderly (65 years or older). % DNA in tail was significantly higher in severely ill patients in both under and over 65 years age subgroups (16.07 ± 1.58% and 14.90 ± 1.51%, respectively) compared to moderate subgroups (9.94 ± 1.40% and 8.83 ± 1.15%, respectively) (*p* < 0.05). Significant differences in the level of DNA damage between adults and elderly in moderate and severe groups were not identified (*p* > 0.05) (Figure 3).

### 2.3. Relationship of DNA Damage with COVID-19 Severity

In order to determine the potential prognostic value of DNA damage for the severity of COVID-19, the correlations of DNA damage with demographic, clinical, and laboratory parameters were analyzed. DNA damage was shown to be positively correlated with INR and creatinine in COVID-19 patients with slight differences by sex and disease severity. Table 5 provides selected correlations that showed significant effects. The results showed that DNA damage was positively correlated with INR (r = 0.471; *p* < 0.001) and creatinine (r = 0.326; *p* < 0.05) in the overall group of patients. A positive correlation of DNA damage with INR (r = 0.398; *p* < 0.001) and creatinine (r = 0.379; *p* < 0.05) was also shown in the total severe group. In the total moderate group (r = 0.456; *p* < 0.05), as well as in the subgroup of moderately ill male patients (r = 0.600; *p* < 0.05), DNA damage was correlated with INR. DNA damage correlated positively with INR in the subgroups of moderately ill females (r = 0.570; *p* < 0.05) and with creatinine in severely ill females (r = 0.534; *p* < 0.05).

### 2.4. Analysis of Prognostic Values of Indicators of COVID-19 Severity

**Figure 4:**
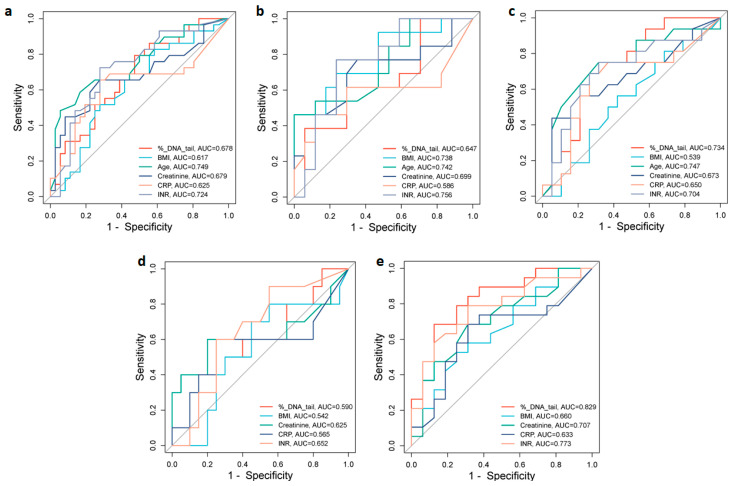
ROC curve analyses for distinguishing moderate from severe groups of COVID-19. ROC curves and AUC values for the (**a**) total group of patients, (**b**) male and (**c**) female patients, (**d**) under 65-year-old, and (**e**) over 65-year-old patients. Figures were obtained using SRplot [available at: http://www.bioinformatics.com.cn/srplot (accessed on 5 August 2024)].

To assess the potential value of different biomarkers for discrimination between moderate and severe COVID-19 patients, we analyzed the area under the receiver operating characteristic (ROC) curves (AUC) of % DNA in tail, age, BMI, CRP, creatinine, and INR, which were significantly increased in the severe group (Figure 4). In the total group of patients, ROC analysis showed that the best predictors of severity were age (AUC = 0.749) and INR (AUC = 0.724). Creatinine, DNA damage, and CRP with corresponding AUCs of 0.679, 0.678, and 0.625, respectively, were also indicative of severity with various levels of predictive power (Figure 4a). The optimal cut-off values that predicted disease severity in the ROC curve were 72.50 years for age, 1.46 s for INR, 78.0 µmol/L for creatinine, 9.72% DNA in tail for DNA damage, and 50.0 mg/L for CRP (Table 6).

In male patients, the INR (AUC = 0.756), age (AUC = 0.742), and BMI (AUC = 0.738) were the strongest predictors of disease severity. CRP (AUC = 0.586) was a less sensitive predictor of disease severity (Figure 4b). The optimal cut-off values were 1.39 s for INR, 72.50 years for age, 26.37 kg/m^2^ for BMI, and 50.0 mg/L for CRP (Table 6). While in female patients the AUC of age (0.747), % DNA in tail (0.734), and INR (0.704) demonstrated association with disease severity (Figure 4c). The optimal cut-off values were 69.50 years for age, 7.96% DNA in tail for DNA damage, and 1.45 s for INR (Table 6).

In patients over 65 years, the AUC of % DNA in tail (0.829) and INR (0.773) demonstrated association with disease severity (Figure 4e). The optimal cut-off values were 12.82% DNA in tail for DNA damage and 1.46 s for INR (Table 6).

## 3. Discussion

Currently, the Omicron variants of SARS-CoV-2 rarely induce life-threatening conditions. However, available prognostic markers to predict potential disease complications are needed to enable earlier intervention and combat future pandemics. Despite numerous studies, prognostic biomarkers for the severity and complications of COVID-19 disease remain poorly understood. The available studies are extremely heterogeneous due to differences in COVID-19 symptoms and severity, sample size, methods used, and a number of other reasons, so interest in this topic remains high. Current studies of predictive biomarkers for COVID-19 have mainly focused on clinical and laboratory parameters [25], with much less attention paid to DNA damage in infected individuals.

Therefore, the main aspect of our study was to analyze DNA damage in infected patients together with demographic and laboratory parameters and to define the biological markers that are useful in predicting a severe disease course in COVID-19 patients. The patients selected for our study were hospitalized with moderate to severe COVID-19; none were admitted to intensive care, and all eventually recovered after treatment. In addition, the patients with moderate to severe COVID-19 did not differ in terms of comorbidities, which are a major factor in COVID-19 complications. Patients were stratified according to sex and age, which are known risk factors for the severity of COVID-19 [26,27]. The current study confirms that COVID-19 patients compared to controls and patients with severe compared to moderate infection have increased DNA damage in blood cells evaluated by the comet assay.

It should be noted that in the control group the levels of DNA damage (median 5.1%, IQR 4.0–5.6%) were in the range of baseline levels of DNA damage previously identified by the hCOMET project (median 4.5%, IQR 1.6–9.9%) [28].

DNA damage was significantly higher in males than in females in a moderate group of patients. Existing studies of DNA damage do not stratify COVID-19 patients by sex, but our data are consistent with studies indicating that, at all ages, males appear to have a higher likelihood of progressing to severe COVID-19 than females [29]. Sex-based behavior, genetic and hormonal factors, and sex differences in biological pathways associated with SARS-CoV-2 infection are considered as possible causes [30]. Moreover, it was suggested that androgens are positive regulators of *TMPRSS2*, which is a key regulator of SARS-CoV-2 entry [31]. No significant differences were found in the level of DNA damage between adults and the elderly in both moderate and severe groups, possibly due to insufficient age differences to influence this parameter (62 (44.5–69.5) versus 72 (63.0–70.0) years). Previous studies using different techniques to measure DNA damage (comet assay and micronucleus test) have shown that high levels of genetic damage are more pronounced in COVID-19 patients [13,15,16,32], as well as in the severe group compared to the moderate group [14,17]. Levels of DNA damage in the total group of patients as well as in subgroups stratified by COVID-19 severity were shown to be correlated with INR (coagulation biomarker) and creatinine (renal injury biomarker) in most cases.

The correlation of comets with creatinine was found to be unreliable in the group of patients studied by Mihaljevic et al. [13]. Although there are no other studies linking DNA damage and renal pathology in COVID-19, there is evidence of increased genomic damage in lymphocytes from patients with uremia, identified by comet analysis [33]. Moreover, comet levels have been shown to be associated with chronic kidney disease in patients with elevated creatinine [34]. The authors link this to impaired DNA repair caused by the uremic state, as well as chronic inflammation associated with increased generation of reactive oxygen species [34]. There are also no studies linking DNA damage to INR, but Basaran et al. [14] showed a correlation of comets with fibrinogen, another coagulation marker in COVID-19 patients.

To date, a large body of literature has accumulated on the mechanisms of DNA damage in cells or organisms infected with a virus. These studies support the important role of DNA damage as a marker of coronavirus infection. Many RNA viruses can induce significant DNA damage in host cells, even when viral replication occurs exclusively in the cytoplasm, as in the case of SARS-CoV-2 [35,36]. DNA damage can contribute to the pathogenesis of RNA viruses [35].

Pánico et al. [37] summarize coronavirus-induced genomic instability and cell cycle deregulation during their replication in mammalian cells. The authors focus on direct mechanisms of DNA damage by coronaviruses through protein–protein interactions between the proteins encoded by the SARS-CoV-2 and human proteins relevant to DNA repair mechanisms and indirect mechanisms via aberrant inflammation, immune response, and oxidative damage.

Besides DNA damage, SARS-CoV-2 was reported to affect two other aspects of the host genome. First, accelerated telomere shortening was found in infected Vero E6 cells [20] and COVID-19 survivors [38]. Second, the virus genome [39] and vaccine mRNA [40] were reported to be reverse-transcribed and integrated into the host genome. Integrated into the genome, SARS-CoV-2 RNA was shown to be expressed as viral–cellular chimeric transcripts in infected cultured cells and patient-derived tissues (lung/heart/brain/stomach) [39].

Chen et al. [41] summarize the alternations of different nuclear pathways caused by SARS-CoV-2, which the authors consider to be understudied. Interestingly, some SARS-CoV-2 proteins are detected in the nucleus, such as NSP1, NSP5, NSP9, and NSP13 [42].

Another mechanism of virus-induced genomic instability is transcriptomic profiling, which was found in the heart tissues of patients with SARS-CoV-2. The most predominant gene sets upregulated in COVID-19 were DNA break, damage and repair, cellular abnormality, and cell cycle, including checkpoints and signaling. The DNA damage present in the SARS-CoV-2 patient samples was further confirmed by nuclear γ-H2AX signals [19].

The monocytes of COVID-19 patients have been shown to release reactive oxygen species capable of inducing DNA damage and apoptosis in neighboring cells. Accordingly, the presence of DNA damage in up to 50% of peripheral blood mononuclear cells and T-cell apoptosis was observed in most patients, which can be considered a cause of lymphopenia [43].

Gioia et al. [12] showed that SARS-CoV-2 causes DNA damage and alters DDR, as observed in cell lines, primary human cells, and in SARS-CoV-2-infected mice and patients with COVID-19, via two mechanisms. One is by affecting cellular dNTP metabolism, leading to DNA replication impairment, and the other is by inhibiting a component of DNA DSB signaling and repair activation, p53 binding protein 1 (53BP1), and reducing DNA repair.

The results of our study indicate that patients with severe COVID-19 are older than the moderate group, and both moderate and severe patients are obese. Both age [26,44] and BMI [45] are generally accepted COVID-19 risk factors. The findings of our study indicate that severe COVID-19 patients have higher INR (coagulation biomarker), creatinine (renal injury biomarker), CRP and PCT (inflammation biomarkers), and fibrinogen (coagulation biomarker) compared to the moderate group. Previous studies have also shown associations of INR [46,47], creatinine [48], CRP [49], PCT [50], and fibrinogen [51,52] with COVID-19 severity.

Several systematic reviews and meta-analyses confirmed the predictive value of inflammatory markers, especially CRP and PCT, in COVID-19 [53,54,55]. Pal et al. [56] suggest that CRP and PCT should be included in clinical practice guidelines to prognosticate COVID-19 cases. According to Kay et al. [57], inflammation and genomic instability are related, so that “inflammation contributes to mutagenesis through the production of reactive oxygen and nitrogen species that can damage DNA, and DNA damage can also exacerbate inflammation”. Mitochondrial or nuclear DNA damage and the DNA damage response represent the common features of every type of inflammation, whether systemic or organ-specific [58]. There is also a study showing the simultaneous increase in CRP and PCT and DNA damage in COVID-19 patients [17].

PCT was shown to be higher in moderate males than in moderate females. Our data are consistent with the study of sex differences in COVID-19 patients, which showed that procalcitonin, among other indicators, was significantly higher in male than in female patients [29].

Biomarkers that were found to differ significantly between moderate and severe forms were evaluated for their predictive value for the severity of COVID-19. Based on the findings of our study, DNA damage, demographic characteristics (age, sex, and BMI), and laboratory parameters (CRP, INR, and creatinine) could identify people at high risk of severe COVID-19.

DNA damage has been shown to be a prognostic indicator for the total group, females and older patients. The prognostic value of age and INR were shown in the total group, males and females. CRP was a prognostic parameter in the total group and in males, while BMI was found to be a prognostic factor only in males. Our results are consistent with previous studies confirming cut-off values of 1.12 mg/dL (99.01 µmol/L) for creatinine [48], 33.55 mg/L [54], 41.8 mg/L [59], 68.15 mg/L [60] for CRP, and 1.075 s for INR [60]. Pijls et al. [61] showed a clear distinction between patients aged more or less than 70 years. A cut-off of 30 kg/m^2^ was shown for BMI [62].

### Limitations

The present study has several limitations. It has a retrospective design. Data were collected from hospital records according to a number of criteria, which carries a risk of selection bias. This study includes a limited number of patients who are unlikely to reflect the full spectrum of COVID-19 disease. The single-center nature of this study limits the ability to generalize the results to the whole population. Therefore, the study sample is not fully representative of COVID-19 patients hospitalized in Armenia during the study period. Despite these limitations, the results of the present study add value to the development of prognostic markers of COVID-19 severity, particularly with regard to DNA damage in infected individuals.

## 4. Materials and Methods

### 4.1. Study Participants

This study included 65 patients, 36 with moderate and 29 with severe SARS-CoV-2 infection, confirmed by real-time polymerase chain reaction. The studied patients were hospitalized at the National Center for Infectious Diseases (Ministry of Health of the Republic of Armenia) during the period of October 2022 to May 2023. At this time, Omicron sub-lineages were predominantly responsible for most of the diagnosed COVID-19 cases in Armenia. Only patients who were not vaccinated prior to infection with COVID-19 were selected for this study. The diagnosis and classification of COVID-19 were based on the Living Guidance for Clinical Management of COVID-19 issued by the WHO [63]. Moderate COVID-19 was diagnosed in patients with pneumonia (fever, cough, and dyspnea) but with oxygen saturation ≥ 90% on room air. The presence of pneumonia (fever, cough, and dyspnea) together with a respiratory rate > 30 breaths/min, severe respiratory distress, or oxygen saturation < 90% on room air was defined as severe COVID-19.

Demographic, clinical, and laboratory characteristics collected at hospital admission were obtained from electronic medical records.

The control group consisted of 24 healthy volunteers with similar demographic and comorbidity characteristics. All participants were informed of the purpose of this study, and their written consent was obtained. The study protocol was approved by the Ethics Committee of the Institute of Molecular Biology of the National Academy of Sciences of the Republic of Armenia (IRB/IEC: IRB00004079, IORG 0003427; approval code # 07/2022; date of approval 30 September 2022).

### 4.2. Blood Sample Collection

Heparinized blood samples were collected from hospitalized COVID-19 patients prior to treatment and healthy donors by venipuncture in the same period of time for laboratory testing and analysis of DNA damage.

### 4.3. Demographic, Clinical, and Laboratory Parameters

Age and body mass index (BMI) were recorded at the time of admission to the hospital. Several comorbidities were considered, although, because of the small frequencies, only some of them were reported. The laboratory parameters were analyzed in the laboratory diagnostic service of the National Center for Infectious Diseases (Ministry of Health of the Republic of Armenia) using standard, accepted methods.

The selected laboratory parameters that were analyzed for the purpose of this study were: white blood cells (WBC), neutrophils (NEU), lymphocytes (LYM), neutrophil to lymphocyte ratio (NLR), platelets (PLT), C-reactive protein (CRP), procalcitonin (PCT), international normalized ratio (INR), activated partial thromboplastin time (APTT), alanine transaminase (ALT), and aspartate transferase (AST). The length of hospitalization was also taken into account.

### 4.4. DNA Damage Analysis

The alkaline single-cell gel electrophoresis (comet) assay was performed to determine DNA damage [64]. Whole blood from patients or healthy volunteers (20 µ) was mixed with 80 µL of low melting point agarose (1%), loaded on slides precoated with normal melting point agarose (1%) and covered with coverslip. Slides were then left to solidify at 4 °C for 10 min. After solidification, slides were immersed in a cold lysing solution (2.5 M NaCl, 100 mM EDTA, 10 mM Tris, 1% Triton X-100, pH 10) for 1 h at 4 °C. After that, the slides were kept for 20 min in freshly made electrophoresis buffer (300 mM NaOH and 1 mM EDTA, pH 13) to allow the unwinding of DNA. Electrophoresis was carried out for 20 min at 25 V (1 V/cm) and 300 mA. Afterwards, the slides were washed in Tris (0.4 M, pH 7.4), and DNA was stained with ethidium bromide (20 µg/mL).

Images of 50 randomly selected cells from each triplicate slide (150 comets per sample) were examined at 400× magnification using a fluorescent microscope (Euromex IS.3153 PLi/6, Arnhem, The Netherlands). Images of comets were analyzed using the Comet Assay IV analysis software (Version 4.3, Perceptive Instruments, Haverhill, Suffolk, UK). % DNA in tail was used to quantify the DNA damage as the most reliable parameter covering the widest range of damage and linearly related to DNA break frequency [65].

### 4.5. Statistical Analysis

Statistical analyses were performed using STATGRAPHICS Centurion 16.2 (Stat-Point Technologies, Inc.; Warrenton, VA, USA) and SPSS version 19 (SPSS, Inc., an IBM Company, Chicago, IL, USA). The Kolmogorov–Smirnov test was used to determine the normality of the distribution. Variables with a non-parametric distribution of data were compared using the Mann–Whitney U test, and parametric data were compared using Student’s *t*-test (two-tailed). Receiver operating characteristic (ROC) of area under the curve (AUC) was used to analyze cut-off values of studied parameters for prediction of severity of COVID-19. The optimal cut-off values were determined based on the maximal value of the Youden’s J index. ROC figures were generated using SRplot (available online: http://www.bioinformatics.com.cn/plot_basic_one_or_multi_ROC_curve_plot_106_en, accessed on 5 August 2024), an online platform for data analysis and visualization [66]. The non-parametric Spearman’s correlation test was used to assess the relationship between the parameters studied. A *p*-value < 0.05 was considered statistically significant.

## 5. Conclusions

The obtained data highlight the potential significance of DNA damage as a biomarker in predicting the severity of COVID-19. Our analysis revealed that patients with severe COVID-19 exhibited higher levels of DNA damage compared to those with moderate disease, with male patients showing more pronounced damage than females. Key predictors of severity included age, CRP, creatinine, and INR, all correlating positively with DNA damage. ROC analysis further validated and identified specific cut-off values for the age (72.5 years), INR (1.46 s), creatinine (78.0 µmol/L), DNA damage (9.72%), and CRP (50.0 mg/L) as significant predictors of severe COVID-19, highlighting their clinical relevance. The cut-off points of the indicators studied are not valid in all subgroups stratified by sex and age, and there are also minor differences in level between them, which is consistent with the importance of sex and age in the severity of infection and should be taken into account. Thus, in the studied group of patients, the levels of DNA damage are well consistent with the severity of COVID-19. On the other hand, it is noteworthy that many of the laboratory parameters included in this study did not show any relation to the severity of the infection. Thus, the inclusion of DNA damage measurement in COVID-19 management protocols along with clinical and laboratory indicators could improve prognostic accuracy and patient care, which warrants further research to validate its utility across diverse populations and clinical settings.

## Figures and Tables

**Table 1:** Demographics and clinical characteristics of COVID-19 patients.

Characteristics	Moderate Group (n = 36)	Severe Group (n = 29)	Control Group (n = 24)
Age, median (IQR)	62 (44.5–69.5)	72 (63.0–70.0) *	64.5 (54.5–72.5)
Male, n (%)	17 (47.2)	13 (44.8)	10 (41.6)
Female, n (%)	19 (52.8)	16 (55.2)	14 (58.4)
Smoking status, n (%)	13 (36.1)	7 (24.1)	8 (33.3)
Alcohol consumption, n (%)	0	1 (3.4)	0
BMI, median (IQR)	26.1 (22.5–29.5)	29.1 (25.3–31.6)	23.0 (22.0–24.5) ^a^
**Comorbidities**			
Hypertension, n (%)	17 (47.2)	19 (65.5)	13 (54.2)
Diabetes, n (%)	11 (30.5)	11 (37.9)	8 (33.3)
Heart diseases, n (%)	6 (16.6)	7 (24.1)	5 (20.8)
Stroke, n (%)	3 (8.3)	0	0
Pneumocystis pneumonia, n (%)	0	2 (6.9)	0
Bronchial asthma, n (%)	2 (5.5)	0	0
Thyroid disease, n (%)	0	2 (6.9)	1 (4.1)
Chronic obstructive pulmonary disease, n (%)	0	4 (13.8)	0
Varicose veins in the lower extremities, n (%)	1 (2.7)	0	1 (4.1)
Epilepsy, n (%)	0	1 (3.4)	0
Gastrointestinal bleeding, n (%)	0	1 (3.4)	0

Data expressed by median (IQR) and numbers (n) (%) were compared by the Mann–Whitney and Student’s *t*-tests (two-tailed), respectively. BMI, body mass index; IQR, interquartile range. * *p* < 0.05 significant difference compared to moderate patients; ^a^ *p* < 0.05 significant difference compared to both moderate and severe COVID-19 patients.

**Table 2:** Laboratory parameters and length of hospital stay of moderate and severe groups of patients with COVID-19 upon admission.

Laboratory Parameters	Reference Values	Moderate Group (Median (IQR))	Severe Group (Median (IQR))
WBC, ×10^9^/L	3.50–10.00	6.18 (4.96–9.25)	9.04 (4.52–13.40)
NEU, ×10^9^/L	1.60–7.00	3.75 (2.83–5.68)	4.34 (2.43–9.94)
LYM, ×10^9^/L	1.00–3.00	1.37 (0.73–2.13)	0.88 (0.63–1.71)
NLR	0.88–4.0	3.19 (1.82–5.40)	3.40 (1.86–9.98)
PLT, ×10^9^/L	150–400	269.00 (221.00–339.00)	246.50 (217.00–328.00)
CRP, mg/L	<5	34.00 (7.20–85.00)	98.50 (61.00–181.00) *
PCT, ng/mL	<0.05	0.42 (0.32–0.46)	0.33 (0.21–0.45)
INR, s	0.85–1.2	1.34 (1.16–1.70)	1.75 (1.44–2.08) *
APTT, s	25–43	39.05 (36.00–42.00)	40.50 (33.50–47.50)
Fibrinogen, mg/dL	200–400	500.00 (442.00–549.00)	500.00 (435.50–501.00)
Creatinine, µmol/L	53–115	48.00 (35.00–64.00)	63.50 (40.00–93.90) *
ALT, IU/L	0–38	23.50 (15.00–30.00)	25.00 (17.00–54.00)
AST, IU/L	0–41	21.55 (15.00–32.00)	22.00 (16.00–41.00)
LOS, days	-	8.00 (7.00–10.00)	13.00 (6.00–20.00) *

* *p* < 0.05—significant difference compared to moderate patients. Data are presented as the median (IQR) and compared by the Mann–Whitney U test. WBC, white blood cells; NEU, neutrophils; LYM, lymphocytes; NLR, neutrophil to lymphocyte ratio; PLT, platelets; CRP, C-reactive protein; PCT, procalcitonin; INR, international normalized ratio; APTT, activated partial thromboplastin time; ALT, alanine transaminase; AST, aspartate transferase; LOS, length of hospital stay.

**Table 3:** Laboratory parameters and length of hospital stay in male and female patients with COVID-19.

Laboratory Parameters	Male		Female	
	Moderate (Median (IQR)) (n = 17)	Severe (Median (IQR)) (n = 13)	Moderate (Median (IQR)) (n = 19)	Severe (Median (IQR)) (n = 16)
Age, years	61 (19–69)	71 (59–81) *	66 (54–70)	73 (67–77) *
BMI, kg/m^2^	24.9 (22.5–26.38)	27.25 (25.3–30.0) *	28.65 (22.6–34.2)	29.3 (25.3–33.7)
WBC,×10^9^/L	6.19 (4.77–10.18)	9.04 (4.52–10.4)	6.18 (5.3–7.09)	9.56 (4.76–13.4)
NEU, × 10^9^/L	4.41 (2.89–8.02)	4.76 (2.56–8.66)	3.59 (2.83–4.61)	3.91 (2.35–10.31)
LYM, × 10^9^/L	1.42 (0.62–1.95)	1.08 (0.6–1.62)	1.37 (0.91–2.13)	0.77 (0.64–1.96)
NLR	3.88 (1.69–8.34)	4.12 (2.24–9.0)	3.05 (1.81–4.75)	3.37 (1.32–14.93)
PLT, × 10^9^/L	257 (243–320)	232 (191.0–295.5)	261.50 (219–298)	240.5 (233–313)
CRP, mg/L	25.5 (6.4–85.0)	179.14 (61.0–213.0) *	41 (9.0–87.0)	87.5 (66.0–122.0)
PCT, ng/mL	0.44 (0.43–0.46) ^a^	0.44 (0.27–0.59)	0.32 (0.28–0.40)	0.28 (0.19–0.34)
INR, s	1.3 (1.22–1.7)	1.56 (1.41–2.05)	1.41 (1.16–1.63)	1.8 (1.47–2.12) *
APTT, s	40 (36.3–42.0)	42.3 (36.5–50.6)	37.9 (36.0–41.9)	37.9 (33.0–43.0)
Fibrinogen, mg/dL	470 (419–500)	500 (439–500)	500 (442–556)	500 (432–515)
Creatinine, µmol/L	50 (44–64)	64 (60–86)	40.5 (32.5–58.5)	62 (35–95)
ALT, IU/L	26.5 (14.9–41.5)	28 (13–65)	20 (15–30)	22 (17–31)
AST, IU/L	27.5 (18.0–32.5)	30 (16–59)	19 (15–25)	21 (16–36)
LOS, days	8.0 (7.0–9.5)	11.5 (5.5–21.0)	8.0 (6.0–10.0)	14 (10–16) *

* *p* < 0.05—significant difference compared to moderate patients; ^a^ *p* < 0.05—significant difference compared to moderate females. For abbreviations and reference values, see Table 2.

**Table 4:** Laboratory parameters and length of hospital stay in COVID-19 patients of different age groups.

Laboratory Parameters	Age Group < 65 Years (n = 30)		Age Group ≥ 65 Years (n = 35)	
	Moderate (Median (IQR)) (n = 20)	Severe (Median (IQR)) (n = 10)	Moderate (Median (IQR)) (n = 16)	Severe (Median (IQR)) (n = 19)
BMI, kg/m^2^	13.89 (7.21–17.42)	27.25 (25.0–30.0)	27.6 (25.1–29.5)	29.3 (26.44–32.0)
WBC, ×10^9^/L	6.07 (4.73–9.61)	10.18 (5.7–17.58)	6.36 (5.16–8.92)	8.33 (4.42–10.99)
NEU, ×10^9^/L	3.56 (2.26–5.68)	6.69 (3.03–10.51)	4.13 (3.27–5.88)	3.91 (2.35–9.26)
LYM, ×10^9^/L	1.56 (1.14–1.76)	1.62 (1.28–1.74)	1.08 (0.56–2.15)	0.74 (0.6–1.48)
NLR	2.28 (1.51–5.06)	4.12 (1.31–10.11)	3.38 (2.60–8.16)	3.37 (2.2–9.85)
PLT, ×10^9^/L	269 (237.5–310.5)	251 (218.5–316.5)	258 (215–310)	245 (218–300)
CRP, mg/L	21.5 (6.0–73.5)	87.5 (50.0–179.14)	41.0 (17.0–87.0)	99.0 (61.0–206.0) *
PCT, ng/mL	0.42 (0.36–0.45)	0.27 (0.19–0.51)	0.38 (0.28–0.47)	0.34 (0.21–0.45)
INR, s	1.41 (1.10–1.77)	1.49 (1.41–2.05)	1.3 (1.16–1.63)	1.81 (1.46–2.09) *
APTT, s	37.9 (34.8–41.9)	46.6 (37.2–55.2)	40.0 (36.0–44.0)	38.8 (32.6–42.3)
Fibrinogen, mg/dL	475.5 (392.0–500.0)	432.0 (419.0–478.0)	522.0 (459.0–606.0)	501.0 (500.0–538.0) ^a^
Creatinine, µmol/L	46 (37–60)	63.0 (40.0–95.0)	50 (35–64)	64.0 (40.0–92.8)
ALT, IU/L	25.0 (16.0–30.0)	23.5 (12.4–50.0)	18.0 (15.0–31.0)	25.0 (18.0–54.0)
AST, IU/L	25.0 (16.0–32.0)	20.0 (15.3–34.5)	20.0 (14.0–28.0)	28.9 (17.0–47.0)
LOS, days	7.5 (6.0–9.0)	10.0 (6.0–15.0)	9.0 (7.0–11.0)	17.0 (14.0–35.0) *^a^

* *p* < 0.05—significant difference compared to moderate patients; ^a^ *p* < 0.05—significant difference compared to age group < 65 years. For abbreviations, see Table 2.

**Table 5:** Correlations of DNA damage with laboratory parameters in COVID-19 patients.

Patient Groups	INR	Creatinine
Total group	r = 0.471; *p* < 0.001	r = 0.326; *p* < 0.05
Total severely affected group	r = 0.398; *p* < 0.001	r = 0.379; *p* < 0.05
Total moderately affected group	r = 0.456; *p* < 0.05	NS
Males	NS	NS
Severely affected males	NS	NS
Moderately affected males	r = 0.600; *p* < 0.05	NS
Females	r = 0.567; *p* < 0.01	r = 0.385; *p* < 0.05
Severely affected females	NS	r = 0.534; *p* < 0.05
Moderately affected females	r = 0.570; *p* < 0.05	NS

NS—non-significant correlation.

**Table 6:** ROC curve analysis of optimal cut-off values, sensitivity, and specificity of indicators of severe COVID-19.

	Cut-Off Point	Sensitivity (%)	Specificity (%)	AUC	*p*-Value	95% CI
**Total group**						
Age, years	72.50	48.3	94.4	0.749	0.001	0.625–0.872
BMI, kg/m^2^	26.44	71.43	52.94	0.617	0.115	0.478–0.757
DNA damage, %	9.72	79.3	52.8	0.678	0.014	0.547–0.809
Creatinine, µmol/L	78.0	46.43	90.32	0.679	0.044	0.541–0.817
CRP, mg/L	50.0	86.36	65.52	0.625	0.003	0.478–0.772
INR, s	1.46	75.00	68.97	0.724	0.004	0.597–0.851
**Male patients**						
Age, years	72.50	46.2	100	0.742	0.025	0.557–0.928
BMI, kg/m^2^	26.37	69.2	76.5	0.738	0.028	0.550–0.925
DNA damage, %	17.79	38.5	94.1	0.647	0.174	0.439–0.855
Creatinine, µmol/L	56.00	76.9	64.7	0.699	0.066	0.495–0.903
CRP, mg/L	50.0	88.89	64.29	0.586	0.038	0.356–0.816
INR, s	1.39	76.9	76.5	0.756	0.018	0.576–0.935
**Female patients**						
Age, years	69.50	75.0	68.4	0.747	0.013	0.572–0.922
BMI, kg/m^2^	29.0	60.0	55.6	0.539	0.691	0.345–0.734
DNA damage, %	7.96	75.0	68.4	0.734	0.019	0.564–0.903
Creatinine, µmol/L	77.00	43.8	94.7	0.673	0.082	0.483–0.863
CRP, mg/L	53.50	68.8	73.7	0.650	0.132	0.452–0.847
INR, s	1.45	75.0	68.4	0.704	0.040	0.519–0.889
**Under 65 years**						
BMI, kg/m^2^	23.4	80.0	45.0	0.542	0.725	0.323–0.762
DNA damage, %	17.99	40.0	85.0	0.590	0.441	0.376–0.804
Creatinine, µmol/L	62.00	60.0	80.0	0.625	0.281	0.417–0.833
CRP, mg/L	50.00	60.0	75.0	0.565	0.580	0.348–0.782
INR, s	1.47	66.66	66.66	0.652	0.370	0.384–0.846
**Over 65 years**						
BMI, kg/m^2^	29.30	52.63	75.00	0.660	0.112	0.475–0.844
DNA damage, %	12.82	68.42	87.50	0.829	0.001	0.686–0.972
Creatinine, µmol/L	78.00	47.36	84.61	0.707	0.192	0.439–0.840
CRP, mg/L	58.00	68.42	68.75	0.633	0.389	0.448–0.818
INR, s	1.46	78.94	66.66	0.773	0.011	0.589–0.926

AUC, area under the ROC curve. For further abbreviations, see Table 2.

## Data Availability

Dataset available upon request from the corresponding authors.
